# The Role of Hematological Parameters in Critically Ill Patients: Mortality Prediction in Elderly Intensive Care Unit Patients

**DOI:** 10.7759/cureus.82183

**Published:** 2025-04-13

**Authors:** Merve Saglam Oz, Burak Toprak, Ridvan Bora, Ismail Demir

**Affiliations:** 1 Department of Internal Medicine, Tosya State Hospital, Kastamonu, TUR; 2 Department of Cardiovascular Surgery, Mersin City Education and Research Hospital, Mersin, TUR; 3 Department of Cardiology, Tarsus State Hospital, Mersin, TUR; 4 Department of Internal Medicine, Izmir Bozyaka Education and Research Hospital, İzmir, TUR

**Keywords:** elderly icu patients, hematological parameters, intensive care outcomes, mortality prediction, rdw and pdw

## Abstract

Background

Elderly patients constitute a high-risk population in intensive care units (ICUs) due to multiple comorbidities, frailty, and limited physiological reserves. Despite advances in intensive care, mortality rates remain significantly high in this demographic. Identifying reliable predictors of mortality is crucial for early intervention and improved clinical outcomes. Hematological parameters, such as red cell distribution width (RDW), platelet-related measurements (including platelet count, mean platelet volume [MPV], and platelet distribution width [PDW]), and inflammatory markers like procalcitonin (PCT), have emerged as potential prognostic indicators. However, their predictive value in critically ill elderly patients remains controversial.

Aims

This study aims to determine whether hematological parameters, including RDW, platelet-related indices (platelet count, MPV, and PDW), and inflammatory markers such as PCT, can predict mortality in elderly patients admitted to ICUs. By establishing their prognostic significance, we seek to enhance early risk stratification and improve clinical decision-making.

Methods

A retrospective observational study was conducted on 238 patients aged 65 years and older who were admitted to the ICU. Laboratory values, demographic data, and clinical severity scores were analyzed. A receiver operating characteristic (ROC) curve analysis and logistic regression analysis were performed to assess the predictive value of hematological markers in relation to 39-day mortality.

Results

Among the study population, mortality occurred in 87 patients (36.6%), highlighting the high-risk nature of the geriatric intensive care cohort. RDW, PDW, MPV, and PCT levels were significantly higher in patients who did not survive (p < 0.0001). The ROC curve analysis identified PCT (area under the curve [AUC]: 0.925, p < 0.0001) and urea (AUC: 0.767, p < 0.0001) as the strongest classifiers of mortality. Logistic regression analysis confirmed PCT (odds ratio [OR]: 1.410, p < 0.0001) and PDW (OR: 1.427, p = 0.013) as independent mortality predictors.

Conclusions

Hematological parameters, particularly RDW, PDW, MPV, and PCT, are strong predictors of mortality in elderly patients in ICUs. These findings emphasize the role of systemic inflammation and coagulation in clinical outcomes within the intensive care setting. Integrating these parameters into clinical risk assessment models may improve early mortality prediction and allow for more targeted management strategies, ultimately improving survival in critically ill elderly patients.

## Introduction

Elderly patients constitute one of the most frequently monitored groups in intensive care units (ICUs). Due to multiple comorbidities and reduced organ reserve capacity, their length of ICU stays tend to be prolonged, and their treatment processes become increasingly complex [1]. The global rise in the elderly population has directly led to an increase in the number of elderly patients requiring ICU care, consequently imposing a greater burden on intensive care services [2]. In geriatric patients, invasive procedures, prolonged mechanical ventilation, and intensive pharmacological treatments are commonly implemented [3].

Mortality rates among elderly patients in ICUs are considerably high, influenced by multiple factors. Increased mortality in this population is primarily due to low nutritional status, diminished functional reserve capacity, presence of underlying pathologies, and reduced regenerative capacity, along with abnormal laboratory findings [4]. Increased mortality in elderly ICU patients is primarily due to low nutritional status, decreased functional reserve, underlying pathologies, and reduced regenerative capacity. Some studies suggest that there is no significant age-related difference in mortality between elderly and younger populations, emphasizing that the primary determinant of mortality is the reason for hospital admission [5]. However, it is well established that conditions such as sepsis, multiple organ failure, and malnutrition significantly contribute to increased mortality rates among elderly patients [6].

The most common reasons for ICU admission among elderly patients include cardiovascular diseases, chronic kidney failure, infections, pulmonary embolism, and acute complications of chronic diseases such as diabetes [7]. Many of these patients are admitted due to advanced-stage diseases, requiring prolonged intensive care support [8]. The management of this patient population necessitates a multidisciplinary approach, and various scoring systems are used to predict mortality [9].

Recent studies suggest that hemogram parameters may serve as significant predictors of mortality in elderly ICU patients [10]. However, traditional scoring systems such as the Acute Physiology and Chronic Health Evaluation II (APACHE II) and Sequential Organ Failure Assessment (SOFA) scores remain the most commonly used tools for mortality prediction. While these scoring systems incorporate multiple clinical and biochemical parameters, their integration with hematological indices has not been well established. Although our primary aim was to investigate hematological parameters, SOFA and APACHE II scores were included to evaluate and control for overall clinical severity and organ dysfunction, thus allowing a comprehensive assessment of independent predictors of mortality. This study aims to explore whether these hematological parameters can complement existing scoring systems to enhance risk stratification in critically ill elderly patients. However, the predictive value of these parameters remains controversial, with conflicting reports in the literature regarding their sensitivity and specificity in different ICU populations. Erythrocyte indices and platelet parameters are particularly associated with inflammatory processes and may play a role in predicting mortality in critically ill patients [11]. Several reports indicate that platelet indices demonstrate a strong correlation with mortality, particularly in the context of acute coronary syndrome and inflammatory conditions [12]. However, studies assessing the impact of these parameters on mortality, specifically in patients aged 65 years and older, remain limited [13].

This study aims to investigate the role of hemogram parameters in predicting mortality among ICU patients aged 65 years and older. Furthermore, we aim to determine whether specific hematological markers can serve as independent predictors of adverse outcomes, thereby facilitating early risk stratification and targeted interventions in critically ill geriatric patients. By evaluating the relationship between mortality and hematologic parameters such as hemoglobin (HGB) level, red cell distribution width (RDW), platelet count, mean platelet volume (MPV), and platelet distribution width (PDW), we aim to contribute to the scientific understanding of intensive care management in the geriatric patient population.

## Materials and methods

Data

Study Design

This study was designed as a retrospective cohort study. The medical records of patients were reviewed retrospectively from the hospital database.

Data Collection

A total of 238 patients aged 65 years and older who were admitted to the Internal Medicine Intensive Care Unit of Sağlik Bilimleri Üniversitesi (S.B.Ü) Bozyaka Training and Research Hospital, İzmir, Turkey, between 2018 and 2020 were included in the study. Patients’ demographic data, including age, gender, APACHE II score, and SOFA score at ICU admission, were recorded. Data were obtained retrospectively from electronic health records and hospital laboratory systems. All variables were clearly defined and measured consistently. Potential confounders were addressed by applying multivariate logistic regression analysis. Additional clinical parameters such as the need for red blood cell transfusion, mechanical ventilation support, polypharmacy exposure, use of central nervous system (CNS)-related medications, and laboratory parameters including complete blood count (CBC) and biochemistry tests were collected.

Exclusion Criteria

Patients diagnosed with hematological malignancies and those without available CBC results within the first 24 hours of ICU admission were excluded from the study. Additionally, patients with active infections, severe hepatic dysfunction, or ongoing immunosuppressive therapy were excluded to minimize confounding factors. Missing data were handled using the multiple imputation method based on chained equations (MICE) to reduce selection bias. Despite these precautions, potential confounders such as nutritional status and preexisting inflammatory conditions could not be fully controlled.

The association between HGB levels, platelet count, RDW, MPV, and PDW with mortality was evaluated. Mortality was assessed within 39 days from ICU admission.

Data analysis

Statistical Analysis

All statistical analyses, including independent samples t-tests and binary logistic regression models, were performed using IBM SPSS Statistics for Windows, version 26.0 (IBM Corp., Armonk, NY). The normality of the data distribution was assessed using the Kolmogorov-Smirnov and Shapiro-Wilk tests. Descriptive statistics were presented as mean ± standard deviation for normally distributed variables, and categorical variables were expressed as frequency and percentage.

Comparisons of categorical variables were performed using Pearson’s chi-square or Fisher’s exact chi-square tests. Independent sample t-tests were used for comparisons between groups (exitus vs. discharged). A receiver operating characteristic (ROC) analysis was conducted to determine the cutoff values of laboratory parameters for predicting mortality. Binary logistic regression analysis was performed to identify the best predictive model and parameters for mortality. A p-value of <0.05 was considered statistically significant. Logistic regression analysis was performed using both the enter and forward stepwise methods. The Nagelkerke R² statistic was calculated to assess the goodness-of-fit and overall accuracy of the logistic regression model. ROC analysis-derived cutoff values were calculated and subsequently applied to logistic regression analysis to classify mortality.

Data availability statement

Datasets generated and/or analyzed during the current study are available from the corresponding author upon reasonable request.

Ethical approval

Ethical approval for the study was obtained from the S.B.Ü. Bozyaka Training and Research Hospital Clinical Research Ethics Committee, with decision number 2021/11, dated 04.11.2021.

Declaration of Helsinki

The study and the writing of the article were prepared in accordance with the Declaration of Helsinki.

Informed written consent

Since this study was retrospective in nature, informed written consent was not required. However, all patient data were anonymized by removing personal identifiers and replacing them with coded identification numbers. Confidentiality was maintained throughout the study process.

## Results

In this study, a total of 320 patients admitted to the ICU were analyzed. Among these, 238 patients were classified as geriatric (≥65 years old). The demographic and clinical characteristics of both the overall patient cohort and the geriatric subgroup were examined, and the relationship between hematological parameters and mortality was investigated.

According to Table 1, the overall mean age of the patients was 71.0 ± 14.0 years, with the youngest patient being 24 years old and the oldest 106 years old. The gender distribution was balanced, with 49.7% of the patients being female (n = 159) and 50.3% male (n = 161). When analyzed by age groups, 55.9% of patients were between 65 and 84 years old, while 18.4% were aged 85 years or older. Among the geriatric patient group (n = 238), the mean age was 77.5 ± 8.3 years, with 50.4% being female (n = 120) and 49.6% male (n = 118). The majority of geriatric patients belonged to the 65-84 age group (75.2%). Regarding red blood cell suspension, 51.6% (n = 165) of the total patient population received blood transfusion, while this proportion was slightly lower at 48.7% (n = 116) among the geriatric subgroup. Analysis of intubation status revealed that 7.2% (n = 23) of all patients required intubation, whereas 7.6% (n = 18) of geriatric patients were intubated. In terms of polypharmacy exposure, 48.3% (n = 115) of geriatric patients were found to be on multiple medications, compared to 40.9% (n = 131) in the overall patient population. Additionally, 28.6% (n = 68) of geriatric patients were receiving CNS-related medications. Survival analysis showed that the overall mortality rate was 35.3% (n = 113), while among geriatric patients, the mortality rate was slightly higher at 36.6% (n = 87) (Table 1).

**Table 1:** Distribution of demographic and clinical data (n = 320) Descriptive statistical analyses were applied to categorical variables, which are presented as frequencies and percentages. Statistically significant values are highlighted in bold. A p-value of <0.05 was considered statistically significant. CNS, central nervous system; Ex, mortality

Variables	Subgroups	All patients (n = 320)	Geriatric patients (n = 238)	Chi-square results
Gender	Female	159 (49.7%)	120 (50.4%)	χ²(1) = 0.01, p = 0.9318, and Cramer's V = 0.00
	Male	161 (50.3%)	118 (49.6%)	
Age classification	18-40	9 (2.8%)	-	χ²(3) = 71.49, p = 0.0000, and Cramer's V = 0.36
	41-64	73 (22.8%)	-	
	65-84	179 (55.9%)	179 (75.2%)	
	≥85	59 (18.4%)	59 (24.8%)	
Red blood cell suspension	Not transfused	155 (48.4%)	122 (51.3%)	-
	Transfused	165 (51.6%)	116 (48.7%)	
Intubation	No	297 (92.8%)	220 (92.4%)	-
	Yes	23 (7.2%)	18 (7.6%)	
Polypharmacy	No	189 (59.1%)	123 (51.7%)	-
	Yes	131 (40.9%)	115 (48.3%)	
CNS medication use	No	246 (76.9%)	170 (71.4%)	-
	Yes	74 (23.1%)	68 (28.6%)	
Survival	Ex	113 (35.3%)	87 (36.6%)	-
	Discharged	207 (64.7%)	151 (63.4%)	

These findings highlight the significant proportion of geriatric patients within the overall ICU population. An increasing trend in mortality was observed with advancing age. Polypharmacy and CNS medication use were more prevalent among geriatric patients, highlighting the need for careful medication management. The low intubation rates suggest that most patients did not require invasive respiratory support during their length of ICU stay. However, the rates of red blood cell transfusion and mortality in geriatric patients remain noteworthy, emphasizing the need for meticulous monitoring both during ICU admission and after hospital discharge. Table 2 demonstrates the baseline clinical severity and ICU characteristics of patients, providing context to interpret the prognostic significance of the hematological parameters studied.

Analysis of the disease severity scoring systems in Table 2 indicates that the SOFA score ranged between 7 and 26, with a mean value of 10.4 ± 2.9, while the APACHE II score varied between 15 and 35, with a mean of 22.9 ± 4.8. A strong correlation was observed between mortality and these scoring systems, as both SOFA and APACHE II scores were significantly higher in non-survivors (p < 0.0001). Conversely, the length of ICU stay did not show a statistically significant difference between patients who survived and those who did not (p = 0.602).

**Table 2:** Clinical and ICU scoring system results (n = 320) Statistical tests applied include the independent samples t-test for continuous variables. Statistically significant values are highlighted in bold. A p-value of <0.05 was considered statistically significant. SOFA, Sequential Organ Failure Assessment; APACHE II, Acute Physiology and Chronic Health Evaluation II; ICU, intensive care unit

Variables	Minimum	Maximum	Mean ± SD	p-value (mortality vs. discharged)
SOFA score	7	26	10.4 ± 2.9	<0.0001
APACHE II score	15	35	22.9 ± 4.8	<0.0001
Length of ICU stay (days)	1	39	7.0 ± 6.5	0.602

In the geriatric patient group, the mean SOFA score was 10.4 ± 2.9, and the mean APACHE II score was 23.1 ± 4.8. The average length of ICU stay in this group was 7.5 ± 6.7 days, ranging from a minimum of one day to a maximum of 39 days. The need for mechanical ventilation was observed in 7.2% (n = 23) of all patients, with the remaining patients being managed without intubation. Among geriatric patients, this proportion was slightly higher at 7.6% (n = 18), indicating an increased requirement for ventilatory support in older individuals compared to younger patients.

Regarding red blood cell suspension, 51.6% (n = 165) of the total patient population received a blood transfusion, whereas 48.4% (n = 155) did not. In the geriatric group, the transfusion rate was slightly lower at 48.7% (n = 116) compared to the overall population.

These findings emphasize the strong association between mortality and SOFA/APACHE II scores, reinforcing that higher scores are indicative of a more severe clinical course and an increased risk of mortality. The lack of a significant difference in ICU length of stay suggests that disease severity, rather than hospitalization duration, is the primary determinant of mortality. Additionally, the higher mechanical ventilation requirement among geriatric patients reflects an increased risk of respiratory failure in elderly individuals. While red blood cell transfusion rates were similar across age groups, the slightly lower transfusion rate in the geriatric subgroup suggests the need for more cautious clinical management strategies in this population.

Table 3 demonstrates significant differences in several laboratory parameters between patients who did not survive and those who were discharged. Although the primary focus of this study is on the geriatric population (patients aged 65 years and older, n = 238), the data presented in Table 3 include all patients admitted to the ICU during the study period (n = 320) to provide a comprehensive comparison of laboratory parameters based on mortality status across the full cohort. Of the 238 geriatric patients, 87 experienced mortality. Hematocrit (HCT) and HGB levels were significantly lower in non-survivors compared to discharged patients (p = 0.020 and p = 0.004, respectively). Neutrophil (NEU) counts were also markedly higher in the mortality group (p = 0.005), indicating an elevated systemic inflammatory response.

**Table 3:** Comparison of laboratory parameters based on mortality status (n = 320) Statistical tests applied include the independent samples t-test for continuous variables. Statistically significant values are marked in bold. A p-value of <0.05 was considered statistically significant. HCT, hematocrit; HGB, hemoglobin; NEU, neutrophil; LYM, lymphocyte; PDW, platelet distribution width; RDW, red cell distribution width; PLT, platelet count; MPV, mean platelet volume; AST, aspartate aminotransferase; ALT, alanine aminotransferase; CRP, c-reactive protein; SEDIM, erythrocyte sedimentation rate; PCT, procalcitonin; ALB, albumin; Ex, mortality

Variables	Ex (n = 113)	Discharged (n = 207)	p-value
	Mean ± SD		
WBC (×10³/µL)	11.8 ± 7.3	10.5 ± 5.6	0.169
HCT (%)	30.5 ± 6.2	32.3 ± 5.3	0.020
HGB (g/dL)	9.7 ± 1.9	10.4 ± 1.7	0.004
NEU (×10³/µL)	10.3 ± 6.1	8.2 ± 4.1	0.005
LYM (×10³/µL)	1.5 ± 2.4	1.4 ± 0.9	0.546
PDW (fL)	15.8 ± 2.0	14.4 ± 2.3	<0.0001
RDW (%)	17.7 ± 3.1	15.9 ± 2.7	<0.0001
PLT (×10³/µL)	179.6 ± 69.0	186.2 ± 62.3	0.448
MPV (fL)	11.3 ± 3.1	10.4 ± 1.2	0.011
HbA1c (%)	6.4 ± 1.4	6.7 ± 1.7	0.270
Urea (mg/dL)	137.5 ± 86.8	79.9 ± 54.5	<0.0001
Creatinine (mg/dL)	11.8 ± 51.0	3.3 ± 13.6	0.132
AST (U/L)	71.4 ± 168.7	53.5 ± 135.9	0.376
ALT (U/L)	28.1 ± 28.3	51.4 ± 159.2	0.085
CRP (mg/L)	133.1 ± 96.4	96.7 ± 77.6	0.002
SEDIM (mm/h)	73.1 ± 32.5	60.9 ± 29.2	0.036
PCT (ng/mL)	20.3 ± 11.8	3.5 ± 4.5	<0.0001
ALB (g/dL)	2.8 ± 2.3	2.8 ± 2.2	0.910
Total bilirubin (mg/dL)	2.5 ± 4.0	1.4 ± 2.9	0.029

Among platelet indices, PDW and RDW levels were significantly higher in non-survivors (p < 0.0001 for both), while MPV values were also notably increased (p = 0.011). Urea levels were substantially elevated in non-survivors compared to discharged patients (p < 0.0001), suggesting renal impairment as a contributing factor to mortality. Inflammatory markers, including CRP and sedimentation rate, were significantly increased in the mortality group (p = 0.002 and p = 0.036, respectively).

Procalcitonin (PCT) levels were markedly higher in non-survivors (p < 0.0001), reinforcing its role as a strong prognostic marker for adverse outcomes. Total bilirubin levels were also significantly elevated in the mortality group (p = 0.029), potentially reflecting hepatic dysfunction in critically ill patients.

The findings highlight the critical role of hematological, inflammatory, and renal function markers in predicting mortality. The lower HCT and HGB levels in non-survivors suggest an association between anemia and poor clinical outcomes. The significantly elevated NEU count and inflammatory markers indicate that systemic inflammation plays a crucial role in mortality risk. Furthermore, the substantially higher urea and bilirubin levels suggest that renal and hepatic dysfunction may be key contributors to adverse outcomes. These results emphasize the importance of early recognition and targeted management strategies in high-risk patients to improve survival rates.

According to Table 4, PCT (area under the curve [AUC]: 0.925, p < 0.0001) was identified as the strongest predictive marker for mortality. A cut-off value of 7.82 ng/mL demonstrated a sensitivity of 93.2% and a specificity of 89.6%, making PCT the most significant prognostic factor for mortality prediction. HCT and HGB levels were significantly lower in the mortality group. The AUC for HGB was 0.651 (p < 0.0001), with a cut-off value of 9.75 g/dL, yielding 66.9% sensitivity and 55.2% specificity for mortality prediction. Among inflammatory markers, RDW and PDW showed significant associations with mortality. The AUC for RDW was 0.687 (p = 0.0009), with a cut-off value of 16.45, 72.7% sensitivity, and 65.7% specificity. Similarly, PDW had an AUC of 0.670 (p = 0.002), with a cut-off value of 15.75, 54.5% sensitivity, and 55.2% specificity. Renal function parameters were also significant predictors of mortality. Urea had a high diagnostic value (AUC: 0.767, p < 0.0001), with a cut-off value of 97.5 mg/dL, 68.2% sensitivity, and 74.6% specificity. Total bilirubin levels were also significantly associated with mortality, with an AUC of 0.707 (p = 0.0002) and a cut-off value of 0.93 mg/dL, 63.6% sensitivity, and 65.7% specificity. Although NEU levels were higher in non-survivors, their predictive value for mortality was lower (AUC: 0.593, p = 0.099). CRP did not show significant predictive value for mortality (AUC: 0.500, p = 0.995), indicating that CRP alone may not be a reliable mortality marker in critically ill patients (Table 4).

**Table 4:** ROC analysis results in geriatric patients (n = 320) Statistical tests applied include ROC analysis to determine the predictive value of different laboratory parameters for mortality. Statistically significant values are marked in bold. A p-value of <0.05 was considered statistically significant. AUC, area under the curve; CI, confidence interval; HCT, hematocrit; HGB, hemoglobin; NEU, neutrophil; PDW, platelet distribution width; RDW, red cell distribution width; MPV, mean platelet volume; CRP, c-reactive protein; SEDIM, erythrocyte sedimentation rate; PCT, procalcitonin; ROC, receiver operating characteristic

Parameter	AUC	Std. error	p-value	95% CI (lower-upper)	Sensitivity (%)	Specificity (%)	Cut-off value
HCT (%)	0.611	0.038	0.004	0.536-0.686	70.2	49.4	29.8
HGB (g/dL)	0.651	0.037	<0.0001	0.578-0.723	66.9	55.2	9.75
NEU (×10³/µL)	0.593	0.056	0.099	0.483-0.703	56.8	65.7	8.88
PDW (fL)	0.670	0.052	0.002	0.568-0.772	54.5	55.2	15.75
RDW (%)	0.687	0.053	0.0009	0.584-0.791	72.7	65.7	16.45
MPV (fL)	0.627	0.055	0.024	0.520-0.734	61.4	56.7	10.35
Urea (mg/dL)	0.767	0.047	<0.0001	0.674-0.860	68.2	74.6	97.5
CRP (mg/L)	0.500	0.056	0.995	0.390-0.609	50.0	44.8	92.3
SEDIM (mm/h)	0.608	0.056	0.054	0.498-0.719	63.6	61.2	66.5
PCT (ng/mL)	0.925	0.029	<0.0001	0.869-0.982	93.2	89.6	7.82
Total bilirubin (mg/dL)	0.707	0.049	0.0002	0.610-0.804	63.6	65.7	0.93

Overall, PCT, urea, and RDW were identified as the most powerful mortality predictors. Particularly, high PCT levels emerged as the strongest prognostic factor. Elevated urea levels, reflecting impaired renal function, also played a crucial role in mortality prediction. Additionally, increased RDW and PDW levels suggest that systemic inflammation and circulatory disturbances significantly impact mortality risk.

These findings highlight the importance of early assessment of specific laboratory parameters in ICU patients, which could play a critical role in mortality prediction. Patients with elevated PCT, urea, and RDW levels should be monitored closely, as early interventions may help reduce mortality rates.

**Figure 1:**
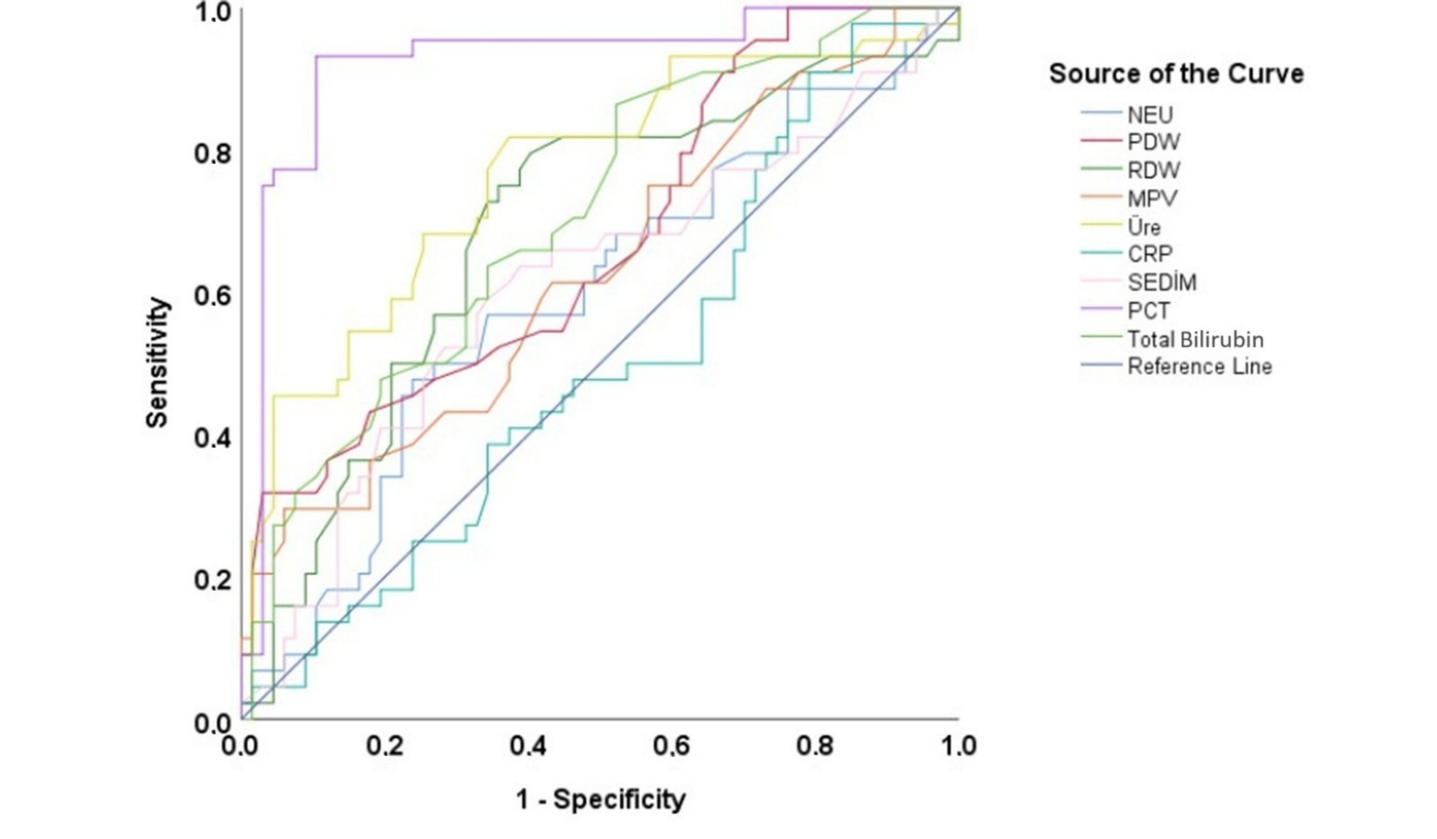
ROC analysis of laboratory parameters significantly associated with mortality in geriatric patients (n = 320) NEU, neutrophil; PDW, platelet distribution width; RDW, red cell distribution width; MPV, mean platelet volume; CRP, c-reactive protein; SEDIM, erythrocyte sedimentation rate; PCT, procalcitonin

According to the ROC analysis results, the highest AUC values for mortality prediction were observed for PCT (AUC: 0.925, p < 0.0001) and urea (AUC: 0.767, p < 0.0001). Additionally, PDW, RDW, and MPV were identified as significant predictors of mortality (Figure 1).

According to Table 5, logistic regression analysis identified PDW, urea, and PCT as statistically significant predictors of mortality. The odds ratio (OR) for PDW was 1.43 (95% CI: 1.1-1.9, p = 0.013), indicating that each unit increase in PDW is associated with a 1.43-fold increase in mortality risk. For urea, the OR was 1.02 (95% CI: 1.00-1.02, p = 0.0003), suggesting that each unit increase in urea levels increases mortality risk by 1.02 times. PCT emerged as the strongest predictor, with an OR of 1.4 (95% CI: 1.3-1.6, p < 0.0001), demonstrating that higher PCT levels significantly increase mortality risk by 1.4 times. This model demonstrated a 92% accuracy in predicting mortality (Table 5). The final logistic regression model, including PDW, urea, and PCT, demonstrated good fit and explanatory power (Nagelkerke R² = 0.723).

**Table 5:** Logistic regression analysis results (n = 320) Variable(s) entered in step 1: HCT, HGB, PDW, RDW, MPV, Üre, PCT, and total bilirubin (model; enter). Variable(s) entered in step 2: PDW, Üre, and PCT (model; forward stepwise). Step 1^a^ represents the initial phase of the logistic regression analysis, where all independent variables were included in the model. Step 2^b^ refers to the final phase, using a forward stepwise method to retain only statistically significant variables. In this step, PDW, urea, and PCT were identified as significant predictors and included in the final model. Logistic regression analysis was applied, and the Wald test was used among statistical tests. A p-value of <0.05 was considered statistically significant, and significant values are indicated in bold. HCT, hematocrit; HGB, hemoglobin; PDW, platelet distribution width; RDW, red cell distribution width; MPV, mean platelet volume; urea, blood urea nitrogen; PCT, procalcitonin; CI, confidence interval; OR, odds ratio

Step	Parameter	B	Std. error	Wald	p-value	OR	95% CI (lower-upper)
Step 1^a^	HCT	0.040	0.114	0.12	0.725	1.041	0.832-1.302
	HGB	-0.123	0.363	0.12	0.734	1.131	0.555-2.304
	PDW	-0.328	0.149	4.86	0.027	1.389	1.037-1.860
	RDW	-0.161	0.085	3.55	0.060	1.175	0.994-1.389
	MPV	-0.192	0.227	0.71	0.399	1.211	0.776-1.891
	Urea	-0.013	0.004	10.04	0.002	1.013	1.005-1.022
	PCT	-0.334	0.050	44.31	<0.0001	1.397	1.266-1.541
	Total bilirubin	-0.025	0.069	0.13	0.718	1.025	0.896-1.173
	Constant	14.598	3.842	14.4	<0.0001	2187682.3	-
Step 2^b^	PDW	-0.355	0.142	6.2	0.013	1.427	1.079-1.886
	Urea	-0.014	0.004	13.4	0.0003	1.015	1.007-1.022
	PCT	-0.343	0.049	48.4	<0.0001	1.410	1.280-1.553
	Constant	10.4	2.394	18.9	<0.0001	33005.7	-

The findings suggest that PDW, urea, and PCT are strong prognostic markers for mortality. The significance of PDW and PCT, both markers of platelet activity and inflammation, highlights the role of inflammatory processes and platelet dysfunction in ICU mortality. Furthermore, the significant impact of urea levels on mortality reinforces the prognostic importance of renal function in critically ill patients.

These results indicate that PDW, urea, and PCT should be considered in clinical risk assessment and management strategies for ICU patients.

## Discussion

In this retrospective study, we examined the impact of hemogram parameters on mortality in ICU patients aged 65 years and older. Our findings indicate that elevated RDW, MPV, and PDW levels are significantly associated with mortality and may serve as important prognostic markers in the geriatric patient population. However, platelet count was not found to be correlated with mortality. This contrasts with prior studies suggesting that a progressive decline in platelet count during hospitalization is associated with poor prognosis, particularly in septic patients. One possible explanation for this discrepancy could be the heterogeneity of our ICU cohort and the relatively short follow-up period. Future studies should focus on dynamic platelet count changes rather than baseline values alone. Previous studies have reported that hemogram parameters are strong predictors of mortality in various diseases [14]. However, research specifically focusing on geriatric patients remains limited, highlighting the need for further studies to expand the existing data.

RDW is a parameter indicating anisocytosis and is widely used in the differential diagnosis of hematologic disorders. However, recent studies suggest that RDW is not only associated with anemia but also with inflammation, metabolic syndrome, and cardiovascular diseases [15]. Increased RDW has been recognized as an independent predictor of mortality in myocardial infarction, stroke, and peripheral artery disease [16,17]. Similarly, in our study, RDW was significantly higher in non-survivors (17.7 ± 3.1) compared to discharged patients (15.9 ± 2.7), with p < 0.0001. This finding supports the hypothesis that RDW is not only a marker of hematologic disorders but also an indicator of systemic inflammation and disease severity in critically ill elderly patients. In the Malmö Diet and Cancer Study, individuals in the highest RDW quartile had a 1.8-fold higher risk of fatal cardiovascular events due to myocardial infarction [18]. A 13.6-year follow-up study of 27,124 individuals without atrial fibrillation found that those in the highest RDW quartile had a 1.33-fold increased risk of developing atrial fibrillation compared to the lowest quartile [19]. In our study, RDW was significantly higher in non-survivors (17.7 ± 3.1) compared to discharged patients (15.9 ± 2.7), with p < 0.0001.

MPV is an indicator of platelet function, and high MPV levels have been associated with an increased risk of thrombosis, myocardial infarction, and cerebrovascular events [20]. These associations suggest that MPV could serve as a surrogate marker for systemic inflammation and endothelial dysfunction, both of which are critical determinants of ICU outcomes. Studies on patients diagnosed with acute coronary syndrome have demonstrated that MPV is directly correlated with long-term mortality [21]. Consistent with these studies, our findings indicate that MPV was significantly higher in non-survivors (11.3 ± 3.1) compared to discharged patients (10.4 ± 1.2), with p = 0.011. This suggests that MPV may be a useful prognostic marker in ICU patients, reflecting increased platelet activation and thrombotic risk. Particularly in acute myocardial infarction, elevated MPV levels indicate platelet hyperreactivity, which worsens prognosis [22]. Similarly, in our study, MPV was significantly higher in non-survivors (11.3 ± 3.1) compared to discharged patients (10.4 ± 1.2), with p = 0.011.

PDW is a marker of platelet heterogeneity, and its elevation is associated with increased platelet activation. Studies have reported that high PDW levels are correlated with atherosclerosis, coronary artery disease, and systemic inflammatory conditions [23]. In studies conducted on acute coronary syndrome patients undergoing primary percutaneous coronary intervention, PDW levels were found to be associated with in-hospital stent thrombosis and long-term major cardiac events [24]. Our study further supports this association, demonstrating that PDW was significantly higher in non-survivors (15.7 ± 2.0) compared to discharged patients (14.2 ± 2.3), with p < 0.0001. These results emphasize the potential role of PDW as a predictor of mortality in critically ill geriatric patients, possibly due to its link with systemic inflammation and platelet activation.

Studies assessing the relationship between platelet count and mortality suggest that baseline platelet count alone may not be sufficient to predict mortality. However, a progressive decline in platelet count during hospitalization is significantly associated with poor outcomes [25]. Particularly in septic patients, a platelet count reduction exceeding 50% has been linked to an increased mortality risk [26]. However, unlike previous reports, our study did not find a significant difference in platelet count between non-survivors (179.6 ± 69.0 × 10³/µL) and discharged patients (186.2 ± 62.3 × 10³/µL), with p = 0.448. This suggests that while platelet function (as reflected by MPV and PDW) may play a role in mortality, absolute platelet count alone may not be a reliable prognostic marker in geriatric ICU patients.

Anemia is a common health issue in the geriatric population, and low HGB levels have been identified as strong predictors of mortality in numerous studies [27]. Research on ICU patients has shown that patients with low HGB levels in the early phase of admission have poorer survival outcomes [28]. In agreement with this, our study found that HGB levels were significantly lower in non-survivors (9.7 ± 1.9) compared to discharged patients (10.4 ± 1.7), with p = 0.004. This finding highlights the impact of anemia on ICU mortality and underscores the importance of early HGB monitoring in critically ill elderly patients. In a 30-month follow-up study on patients with systolic heart failure, anemic patients exhibited significantly higher mortality rates [29]. Consistent with these findings, our study demonstrated that HGB levels were significantly lower in non-survivors (9.7 ± 1.9) compared to discharged patients (10.4 ± 1.7), with p = 0.004.

In conclusion, our study highlights that hemogram parameters play a crucial role in predicting mortality in geriatric ICU patients. Particularly, RDW, MPV, and PDW levels should be considered in clinical management to facilitate the early identification of high-risk patients. These parameters are not only associated with anemia and platelet function but also with systemic inflammation, thrombosis risk, and metabolic processes, emphasizing their multifaceted role in critically ill geriatric patients. In particular, elderly ICU patients presenting with RDW >16.5% and PCT >7.8 ng/mL should be closely monitored for potential deterioration. Clinicians should consider incorporating these parameters into routine ICU assessments to facilitate early interventions and improve patient outcomes.

Limitations of the study

The retrospective nature of the study limits causal inferences and may introduce selection bias. Data were collected from a single center, which may affect the generalizability of the findings. Potential confounding factors such as nutritional status, underlying hematological disorders, and preexisting inflammatory conditions were not fully controlled. The study’s follow-up period was limited to 39 days, which may not fully capture long-term mortality trends. Additionally, the lack of comparison with non-geriatric ICU populations restricts the ability to determine whether these hematological parameters are uniquely predictive in elderly patients or applicable to broader ICU cohorts.

## Conclusions

This study demonstrates that hemogram parameters are strong predictors of mortality in geriatric ICU patients. Elevated RDW, MPV, and PDW were significantly associated with mortality, whereas platelet count was not a significant predictor. These parameters are linked not only to hematological conditions but also to systemic inflammation, cardiovascular diseases, and metabolic disorders. Their accessibility, affordability, and rapid results make them valuable tools for early risk assessment.

Our findings suggest that incorporating RDW, MPV, and PDW into clinical scoring systems may enhance the management of high-risk elderly patients. Particularly, patients with RDW >16.5% and PCT >7.8 ng/mL should be closely monitored. These markers may support timely interventions and better outcomes. Future large-scale prospective studies are warranted to validate these findings across different intensive care settings.
